# Interplay between cofactors and transcription factors in hematopoiesis and hematological malignancies

**DOI:** 10.1038/s41392-020-00422-1

**Published:** 2021-01-20

**Authors:** Zi Wang, Pan Wang, Yanan Li, Hongling Peng, Yu Zhu, Narla Mohandas, Jing Liu

**Affiliations:** 1 Department of Hematology, Institute of Molecular Hematology, The Second Xiangya Hospital, Central South University, 410011 ChangSha, Hunan China; 2 Molecular Biology Research Center and Hunan Province Key Laboratory of Basic and Applied Hematology, School of Life Sciences, Central South University, 410078 Changsha, Hunan China; 3 Red Cell Physiology Laboratory, New York Blood Center, New York, NY USA

**Keywords:** Epigenetics, Haematopoiesis, Haematological cancer

## Abstract

Hematopoiesis requires finely tuned regulation of gene expression at each stage of development. The regulation of gene transcription involves not only individual transcription factors (TFs) but also transcription complexes (TCs) composed of transcription factor(s) and multisubunit cofactors. In their normal compositions, TCs orchestrate lineage-specific patterns of gene expression and ensure the production of the correct proportions of individual cell lineages during hematopoiesis. The integration of posttranslational and conformational modifications in the chromatin landscape, nucleosomes, histones and interacting components via the cofactor–TF interplay is critical to optimal TF activity. Mutations or translocations of cofactor genes are expected to alter cofactor–TF interactions, which may be causative for the pathogenesis of various hematologic disorders. Blocking TF oncogenic activity in hematologic disorders through targeting cofactors in aberrant complexes has been an exciting therapeutic strategy. In this review, we summarize the current knowledge regarding the models and functions of cofactor–TF interplay in physiological hematopoiesis and highlight their implications in the etiology of hematological malignancies. This review presents a deep insight into the physiological and pathological implications of transcription machinery in the blood system.

## Introduction

Hematopoiesis is a complex hierarchical differentiation process that involves the production of hematopoietic stem cells (HSCs) and progenitor cells (HPCs) and their differentiation into terminally differentiated cells. Lineage commitment to hematopoietic cells requires finely tuned regulation of lineage-specific gene expression patterns through the activation of lineage-specific transcriptional programs, and the concomitant suppression of programs related to the early multipotent state or alternative lineages. Hematopoietic transcription factors (TFs) bind directly to regulatory elements to elegantly regulate gene expression by forming transcription complexes (TCs) with cofactors (coactivators and corepressors) and basal transcriptional machinery in a context-dependent manner. TCs are involved in multiple steps of transcription, including preinitiation complex (PIC) formation, post-recruitment processes, DNA loop formation, transcriptional initiation, and elongation.^1^ The normal composition and function of TCs are essential to maintaining HSC self-renewal and normal hematopoiesis and preventing malignant transformation.

Within TCs, cofactors play a pivotal role in modulating TF activity by “reading” the chromatin landscape or preparing the site for binding. Cofactors function in either an enzymatic or nonenzymatic manner. Generally, nonenzymatic cofactors include TBP-associated factors (TAFs) and Mediators (Meds). Enzymatic cofactors can be classified into two main mechanistically distinct groups: (1) histone-modifying cofactors, including histone deacetylase (HDAC), histone acetyltransferase (HAT), histone methyltransferase (HMT), and histone demethylase (HDT) and (2) ATP-dependent chromatin-remodeling cofactors, which are classified by their ATPase subunits into four major families, including the SWI/SNF, ISWI, Mi-2/NuRD, and INO80/SWR1 families.

Normal cofactor–TF interactions or interplay are involved in multiple aspects of hematopoiesis, such as stemness maintenance and lineage commitment. Exome and whole-genome sequencing revealed some specific mutations or translocations that may produce gain/loss of function in cofactors. Deregulation of cofactors by abnormal expression or activity disrupts normal cofactor–TF interplay or facilitates formation of oncogenic TCs, which upsets gene regulatory networks. Therefore, cofactors are frequent targets of genomic alterations in cancer. In recent years, we have gained considerable knowledge about how TFs and chromatin landscapes control gene expression. Attention has now turned to understanding the dynamic and multifaceted interplay between these regulatory layers and how they cooperate to determine gene expression responses to cellular signals. Here, we provide unique insight into the interplay between TFs and cofactors and how dysregulation of their interplay results in aberrant transcriptional regulation programs. We also summarize the cofactors with a high frequency of association with genetic disorders and cofactor-targeting inhibitors in hematological malignancies.

## TBP-associated factors

**Fig. 1:**
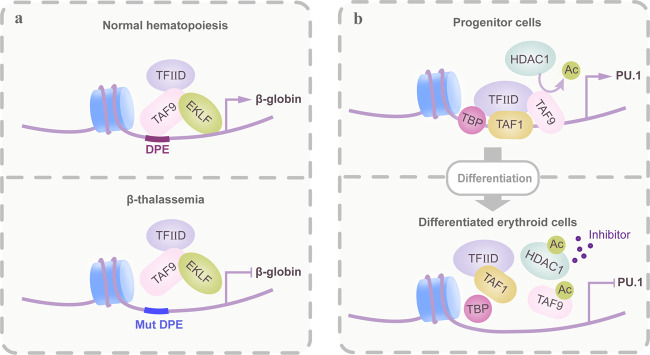
Modes of gene transcriptional regulation by TAF-containing TCs. A deeper insight into the functional importance of TAFs as cofactors that connect transcription complexes with DNA. In addition, other cofactors within one transcription complex could indirectly affect gene transcription via regulating TAFs. **a** As a component of TFIID, TAF9 associated with the transcription factor EKLF provides a platform for the recruitment of TFIID transcription complexes, particularly at promoters that contain the initiator (INI) and DPE box (i.e., β-globin promoter). In disease conditions, β-thalassemia-causing mutations across the binding region disrupt the formation of the transcription complex and thus generally inhibit transcription efficiency. **b** The DNA-binding ability and activity of TAF9 are determined by its acetylation level. During erythropoiesis, TAF9 acetylation gradually increases due to a decrease in HDAC1-associated deacetylase activity, which subsequently results in the disassociation of the TFIID complex and transcriptional repression

Initiation of transcription by RNA polymerase II (Pol II) requires the general TF TFIID to assemble the Pol II PIC. TAFs, as components of TFIID, function in TATA-containing and TATA-less promoter recognition by engaging in direct and selective interactions with transactivators and/or core promoter sequences to facilitate PIC assembly.^2^ The hematopoietic TFs GATA1, EKLF, and NF-E2 have been shown to be associated with TFIID via interaction with TAFs. TAF/TF interactions are critical for dynamic changes in the occupancy of TAF-containing complexes. For example, the TAF10 and GATA1 interaction mediates the recruitment of two TAF10-containing complexes (TFIID and SAGA) to GATA1-responsive promoters and the GATA1 locus itself in mouse fetal erythroid cells. These complexes were significantly less enriched at the same locus in adult erythroid cells due to a reduction in their association. TAF10 ablation in fetal erythroid cells results in a block in erythropoiesis with downregulation of GATA1 and its downstream genes, suggesting that genetic inactivation of TAFs may phenocopy the effects of targeting their interacting TFs.^3^ In some cases, the TAF and TF interaction depends largely on the architecture of the gene promoter. For instance, during erythroid differentiation, TAF9 interacts with EKLF and functionally accentuates EKLF-mediated transcriptional activation of β-globin, in which TAF9 recruitment to the downstream promoter element (DPE) occurs in an EKLF-dependent manner. However, the *AHSP* gene, which does not contain a DPE, although dependent on EKLF for transcription, does not require TAF9.^4^ Specifically, ablation of the TAF9–β-globin interaction by β-thalassemia-causing mutations leads to a decreased level of promoter activity, suggesting that restoration of TAF complex occupancy at hematopoietic genes may be critical for normal hematopoiesis^4^ (Fig. 1). In addition, regulation of TAF9 protein modification is important for TFIID complex stability. In human CD34^+^ cells, deacetylation of TAF9 by HDAC1 is required for TFIID complex recruitment to the PU.1 promoter. Upon erythropoiesis, inactivated HDAC1 is located on the silenced PU.1 promoter, preventing the recruitment of the TAF9–TFIID complex, which leads to PU.1 transcriptional repression^5^ (Fig. 1).

TAFs may guide the localization of oncogenic fusion TFs to promoter sites. In inv(16) acute myeloid leukemia (AML), the CBFβ–MYH11/RUNX1 complex interacts with TAFs and occupies genomic regions that have been implicated in hematopoietic stem cell self-renewal, such as TBP and RNAPII.^6^ The TAF/TF interaction region or promoter recognition domains of TAFs may provide a therapeutic target to disrupt abnormal transcription programs. Indeed, NF-E2 lacking a TAF_II_130-interacting domain loses its ability to support enhancer-dependent transcription of *globin* genes.^7^ Targeting TAF1 bromodomains with the inhibitor BAY-299 produces antiproliferative effects via affecting GATA1 and MYC transcriptional programs in K562 cells.^8^ Furthermore, TAF12, in a heterodimer with TAF4, interacts with the transactivation domain of MYB, supporting transcriptional activation of MYB and protecting it from proteasome-mediated protein degradation. Perturbation of the TAF12/MYB interaction by peptides impairs MYB activity and leads to regression of AML in mice.^9^

## Mediators

The Mediator is a large macromolecular complex that serves as a molecular bridge between enhancer-bound TFs and RNA Pol II, thus facilitating PIC assembly. The Mediator is composed of four separate modules, wherein the head, middle, and tail modules form a core that associates reversibly with a kinase module comprised of Med12, Med13, CDK8, and Cyclin C. The Med1/TRAP220 subunit, a component of the middle module, is involved in multiple hematopoietic lineages via interaction with GATA1, nuclear hormone receptors and IKAROS. Med1 knockdown mice show a specific block in erythroid development with a lack of *β-globin* gene expression but not in myeloid or lymphoid development, suggesting that Med1 has lineage-specific functions.^10^ In erythropoiesis, Med1 interacts with the N-terminal zinc finger of GATA1 and forms a GATA1–Med1–Med17–Pol II complex, which is critical for GATA-1-mediated transactivation.^11^ Through interaction with the nuclear receptors Vitamin D receptor (VDR) and retinoic acid receptor (RAR), Med1 is involved in the differentiation of HPCs toward monopoiesis and myelopoiesis, respectively.^12^ Furthermore, Med1 in stromal cells appears to play an important role in supporting hematopoietic stem and/or progenitor cell (HSPC) growth by upregulating VDR-mediated and Runx2-mediated transcription from the osteopontin promoter.^13^ During pre-B-cell differentiation, IKAROS, in the company of other B cell master regulators, such as Med1, PAX5, EBF1, and IRF4, defines a set of superenhancers (SEs) with a highly permissive chromatin environment that supports transcription of key pre-B-cell differentiation genes.^14^

Med12 contributes greatly to the interaction of TFs with the Mediator complex. In HSCs, Med12 colocalizes with RUNX1, GATA2, and FL1, where it cooperates with additional cofactors, such as P300, LMO2, and TAL1, to maintain hematopoietic gene activity. Med12 deletion causes H3K27Ac depletion at enhancers and failure of hematopoietic-specific transcriptional programs.^15^ Med12 mutations are involved in abnormal hematopoiesis. For instance, a loss of function mutation of Med12 that leads to dissociation of Cyclin C-CDK8/19 from the core Mediator contributes to chronic lymphocytic leukemia (CLL) pathogenesis by activating NOTCH signaling.^16^ Interestingly, several cofactors interacting with Med12 in HSPCs are also frequently mutated in leukemia and lymphoma, including P300/CBP, KMT2D, WDR5, and KMD6A,^17–19^ suggesting that alterations in Med12-dependent enhancer regulation may be a potential pathogenic factor. In zebrafish, Keightley et al. identified a V1046D mutation in Med12 that can cause defects in myelopoiesis without affecting the erythroid lineage, which may be mediated by defective transcription of myc.^20^

The Mediator kinase CDK8 and its paralog CDK19 act as major ingresses of developmental and oncogenic signaling through Mediator. CDK8/19 have been identified as cofactors of a number of TFs, such as c-Jun,^21^ TCF/LEF/β-catenin,^22^ Smads,^23^ HIF1A,^24^ and NF-κB.^25^ CDK8/19-targeted substrate phosphorylation impacts TF activity and the transcription of lineage-controlling TFs. For example, CDK8 inhibitors enhance IL-10 production during innate immune activation in human and mouse primary macrophages and dendritic cells (DCs) via diminished phosphorylation of the c-Jun subunit of the AP-1 transcription complex.^21^ CDK8/19 can also inhibit gene expression of SE-associated lineage-controlling TFs identified in related CD14+ monocytes, including the tumor suppressors IRF1, IRF8, CEBPA, and ETV6, and the kinase activity of CDK8/19 can be pharmacologically targeted as a therapeutic approach to AML.^26^

**Fig. 2:**
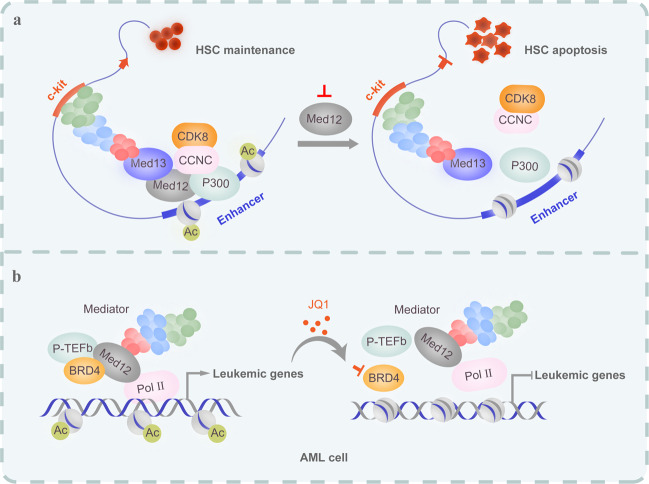
Modes of Mediator-dependent enhancer regulation controlling both normal and malignant hematopoiesis. Generally, Mediator functions as a large coactivator complex that recruits enhancer-localized TFs to the promoter, where some members of the Mediator module cooperate with additional transcriptional cofactors to maintain active enhancers of essential hematopoietic genes. Gain or loss of function mutations of MEDs have been identified to lead to malignant hematopoiesis. To date, no drugs have been developed to target MEDs or their mutation sites. Additionally, bromodomain and extraterminal (BET) proteins, such as BRD4, regulate their downstream target genes, at least in part, by interacting with the Mediator complex and have been suggested as targets of bromodomain inhibitors to treat MED-mediated malignancies. **a** An enhancer-specific role of Med12 in preserving H3K27Ac levels for maintaining the active state of hematopoietic enhancers, such as the c-kit gene, via cooperation with P300. Deletion of Med12 causes H3K27Ac loss at enhancers of essential HSC genes, failure of hematopoietic-specific transcriptional programs and apoptosis in HSCs. **b** BRD4 and Mediator can mutually stabilize one another’s occupancy at cis elements of leukemic genes (AML) bound by acetylated nucleosomes, which promotes the transcriptional elongation of Pol II by facilitating P-TEFb recruitment. The BET bromodomain inhibitor JQ1 causes a dramatic release of Mediator from the leukemia genome, leading to transcriptional suppression of leukemic genes

Meds are involved in pathway-response gene transcription. For example, MAPK/ERK pathway-dependent processes are essential for T cell development. T cells lacking the tail module protein Med23 failed to efficiently populate the peripheral lymphoid organs. Med23 null thymocytes displayed decreased expression of the MAPK-responsive TFs MEF2 and KLF2.^27^ In both T cells and MEFs, Med23 regulates the transcription of T cell transcription factor KLF2 by coactivating MEF2.^27^ Furthermore, individual subunits of Mediator interact specifically with different transcriptional modulators to selectively fine-tune the regulation of specific signaling pathways. For example, the ARC/Med6 complex links TGFβ/Activin/Nodal/Smad2/3 signaling to transcriptional activation by binding to the Smad2/3–Smad4 complex, which recruits the ARC/Med6 complex to Activin/Nodal-responsive promoters, in response to TGFβ.^23^ Furthermore, the transactivation domain of β-catenin interacts with the Med12 subunit in Mediator and activates transcription of Wnt-responsive genes via recruitment of the ARC/Med6 complex.^28^ CDK8 also stimulates Wnt/β-catenin signaling by phosphorylating/inhibiting E2F1, which is a negative regulator of β-catenin-mediated transcription.^22^ Bromodomain and extraterminal protein (BET) inhibitors are suggested to be applicable in targeting Med-mediated malignancies. In this respect, the BET protein BRD4 and the Mediator complex are linked coactivators that maintain MYB-specific transcriptional activation for AML maintenance. The BET inhibitor JQ1 exerts antileukemic effects by releasing the complex from the AML genome^29^ (Fig. 2).

## Chromatin remodeling complexes (CRCs)

**Fig. 3:**
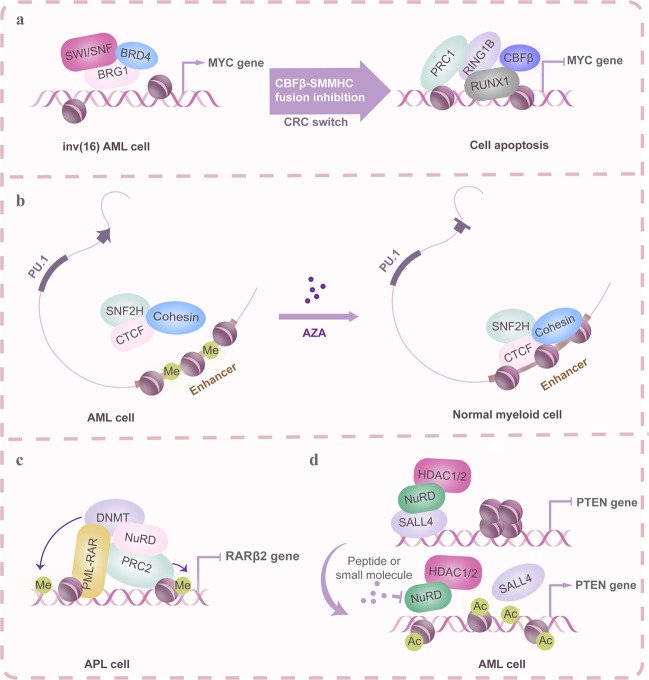
Chromatin remodeling complexes (CRCs) are implicated in malignant hematopoiesis via their interplay with TFs. In many cases, CRCs are indispensable to the oncogenic functions of leukemia-associated and fusion TFs due to their multisubunit properties and/or recruitment of other transcriptional complexes, such as the polycomb repressive complex. In addition to remodeling histone–DNA interactions, epigenetic subunits confer to CRCs the ability to modulate regulatory region activity in the genome by affecting histone modifications or DNA methylation levels. Based on this knowledge, the following two treatment strategies have been suggested: (1) release or transition of CRC occupancy by changing DNA methylation levels or targeting oncogenic TFs. (2) Targeting the interactions between CRCs and oncogenic TFs. **a** The BRG1–SWI/SNF complex together with BRD4 promotes superenhancer (BRD4-dependent MYC enhancer) activity of the MYC gene. After treatment with the CBFβ-SMMHC fusion protein inhibitor, the BRG1–SWI/SNF complex occupancy is replaced by the RUNX1–PRC-repressive complex, which inhibits the MYC transcriptional program. **b** In AML, the binding of the Smarca5/SNF2H-CCCTC-binding factor (CTCF) complex at the PU.1 gene is blocked due to DNA methylation. Upon treatment by AZA-mediated DNA demethylation, the Smarca5/SNF2H complex is recruited to the enhancer of the PU.1 gene and blocks its expression. **c** In APL, the PML-RARα fusion binds and recruits NuRD, the PRC2 complex and DNA methyltransferase 3a (DNMT3a) to the tumor suppressor gene RARβ2, which in turn leads to chromatin compaction and consequent promoter silencing. **d** The oncogenic TF SALL4 promotes leukemogenesis, at least in part, by repressing PTEN gene expression through recruitment of the NuRD/HDAC complex. Disrupting the SALL4 and NuRD/HDAC complex interaction could reverse the repression of the PTEN gene and reduce tumor cell viability

CRCs are ATPase/helicase-dependent remodeling enzymes that serve as ‘molecular motors’ that couple ATP hydrolysis to the perturbation of histone–DNA contacts with respect to individual nucleosome core particles. As multisubunit transcription machineries, they are essential for the transcription of numerous hematopoietic genes (Table 1 and Fig. 3).

**Table 1:** Transcriptional complexes of TAFs, Mediators, and chromatin remodeling-related cofactors in normal and malignant hematopoietic cells and their functions

Co-factors	Complex components	Cell type	Functions of TCs
TAFs	TAF9–EKLF	MEL cells	Enhancing transcriptional activation of EKLF to the *β-globin* gene
	TAF9–HDAC1	MEL, K562 and human CD34+ cells	Deacetylation of TAF9 by HDAC1 is required for PU.1 transcription
	TAF (4/4b, 9, 10, 12) –SAGA or TFIID–GATA1	Mouse and human erythroid cells	Regulation of GATA1 target genes and the autologous control of GATA1 expression
	TAF_II_130–NF–E2	K562 and CB3 cells	Promotion of Enhancer-dependent transcription of *β-globin* and *α-globin* genes
	TAF (1, 3, 4, 5, 6, 7, 9 and 10)–CBFβ–MYH11–RUNX1	ME-1 inv (16) cells	Guiding the localization of CBFβ or the fused CBFβ–MYH11 to promoter sites
	TFIID or SAGA complex–TAF12–TAF4–MYB	Murine AML cells (RN2 cells)	TAF12 facilitates transcriptional activation of MYB and protects it from degradation
Mediators	Med (1, 14, 17)–GATA1	Mouse erythroid leukemia cells	As a cofactor for GATA1 to enhance GATA1-mediated transactivation
	Med1–vitamin D receptor	HL-60 cells	Involvement in the differentiation of hematopoietic progenitor cells into monocytes
	Med1–retinoic acid receptor	HL-60 cells	Involvement in the differentiation of hematopoietic progenitor cells into granulocytes
	Med (12,13)–p300–CDK8–CCNC	HPC-7 cells	The maintenance of the active state of hematopoietic enhancers
	Med23–MEF2	T-cells and MEF cells	MED23 is required for full activity of the MAPK-responsive transcription factor MEF2
	BRD4–Mediators (MED12, 13, 23 and 24)	Mouse MLL-AF9; NrasG12D AML cells	Sustaining expression of BRD4, MYC, and MYB target gene signatures
SWI/SNF	BRG1-containing E-RC1 complex-EKLF	MEL cells	The complex is critical for chromatin remodeling and transcription with EKLF
	BRG1–EKLF–TBP–NF–E2–CBP	Human CD34+ cells	Facilitating chromatin remodeling of the human β-globin promoter and β-globin activation
	BRG1–GATA1–Scl/TAL1–mSin3A–HDAC2	MEL cells	Repression of protein 4.2 promoter activity in an HDAC-dependent manner
	BRG1–BAFs (250A,170, 155, 53A and 47)	hESCs	Regulation of the pluripotency of hESCs by modulating the acetylation levels of H3K27 at the enhancers of lineage-specific genes
	BRG1–BAFs (47, 57, 60a and 170)–PYR complex-NuRD/Mi-2-Ikaros	MEL cells	Facilitating fetal-to-adult globin gene switching, most likely through an effect on higher-order chromatin structure
	BRG1–INI1–RUNX1	Jurkat cells	RUNX1 interacts with BRG1 and INI1 and supports binding of SWI/SNF complex to RUNX1 target genes related to hematopoietic lineage progression
	BRG1–ATF3–β-actin	HL-60 cells	ATF-3 cooperates with BRG1 and β-actin to initiate left-handed Z-DNA formation and subsequently transactivate the *SLC11A1* gene
	C/EBPβ–hBRM–BAF155–Myb	HD3 erythroblasts	Fusion of N terminus of C/EBPβ with Myb conferred hBrm responsiveness to the chimeric transcription factor and enabled it to activate the *mim-1* gene
	BRG1–STAT6–NFAT1	Primary mouse Th cells	BRG1 recruitment to the Th2 LCR depends both on cytokine signals through STAT6, as well as signaling through the TCR via NFAT1
	BAF60B–CEBPε	Human promyelocytic cell line NB4	BAF60B interacts with CEBPε and controls expression of neutrophil proteins stored in specific granules
SNF2H-ISWI	SNF2H–ACF1–GATA1	MEL and G1E cells	NA
	SNF2H–ACF1	Murine EL4T cells, Primary CD4T cells	Involvement of both repression (e.g., *IL-2*) and activation (e.g., *IL-3*) of cytokine genes
	SNF2H–CTCF–Cohesin complex	MEL and OCI-M2 cells	The complex is recruited to the enhancer of *SPI1* gene and block its expression
NuRF-ISWI	Bptf–Snf2L–pRb–Srf	Mice thymocytes	Srf recruits NuRF to the Bptf-dependent genes, which is important for CD4/CD8 TCRβ+ thymocytes
Mi-2/NuRD	MTA2–MBD3–Mi-2–HDAC1/2–PRC2–DNMT3a–PML–RARα	NB4 leukemic cells	Establishment and maintenance of aberrant epigenetic silencing imposed by PML-RARα
	SALL4–MTA2–Mi-2–HDAC1/2	Human ESCs, NB4 cells	Repression of PTEN and SALL1 expression, contributing to self-renewal in ESC and leukemic stem cell
	Mi-2/NuRD–P-TEFb complex–PP1–IKAROS	Lin- HPCs and Jurkat cells	Facilitating transcription elongation of IKAROS-target genes and normal differentiation of hematopoietic progenitor cells
	Mi-2β–p300–HEB	DP thymocytes	Stablizing recruitment of basal transcription factors and causing histone H3-hyperacetylation at the CD4 enhancer
	Mi-2β–MOZ–Ikaros–SWI–SNF	DP thymocytes	Concomitant binding of Mi-2β with Ikaros to the CD4 silencer caused silencer inactivation, thereby allowing for CD4 expression
	Mi-2β–KDM6A–CBP–H3K27Ac	Human primary AML cells	Crosstalk among Mi-2β, KDM6A, H3K27Ac, and CBP toward induction of *DOCK5/8* expression and maintenance of Rac GTPase program in AML cells
	MTA2/NuRD–AIOLOS/IKAROS or MTA2/NuRD–OCA-B	Human pre-B leukemia 697 cells	MTA2 cooperates with AIOLOS/IKAROS and OCA-B to suppress Pre-BCR (B cell receptor) genes during the Pre-B to immature B transition
MTA/NuRD	MTA1/3–RbAp46-containing NuRD complex–BCL11B	Primary human CD4+ T cells, Jurkat cells	Regulation of *IL-2* gene during activation of human CD4+ T
	BCL-6–MTA3-contaning NuRD/Mi-2 corepressor complex	Lymphocyte and plasma cells	Leading to reprogramming of the plasma cell transcriptional program to a B lymphocyte pattern

*NA* not applicable.

### SWI/SNF complex

Human SWI/SNF complexes are heterogeneous mixtures of proteins (∼2 MDa) composed of 12–15 subunits and fall into two broad classes depending on whether they contain one of two ATPases, BRG1 or hBRM. SWI/SNF complexes are implicated in erythroid and myeloid lineage development via association with the hematopoietic TFs EKLF,^30^ Ikaros,^31^ GATA1,^32^ RUNX1,^33^ ATF3^34^ and C/EBPβ^35^ (Table 1). Different complex compositions confer distinct functions to SWI/SNF complexes. For example, in erythroid cells, SWI/SNF-related EKLF coactivator remodeling complex 1 (E-RC1) displays functional selectivity toward EKLF and facilitates EKLF-dependent human β-globin transcription by disrupting the nucleosomes over the promoter. Within the complex, the BAF57 subunit is critical for chromatin remodeling and transcription with EKLF.^36,37^ An Ikaros–SWI/SNF complex called the PYR complex broadly footprints a pyrimidine-rich element between the human γ-globin and β-globin loci. The PYR complex also contains NuRD/Mi-2 complex subunits, which may confer to the PYR complex nucleosome remodeling and HDAC activities whereby it represses the *γ-globin* gene and facilitates globin switching by permitting TFs, such as EKLF, to access adult *globin* genes.^38^

Although SWI/SNF complexes are suggested to antagonize chromatin-mediated repression, they can also associate with corepressors at a subset of genes, leading to transcriptional repression. For example, BRG1 acts as a component of the GATA-1–Scl/TAL1 complex, which inhibits protein 4.2 transcription in erythroid progenitors through recruitment of the corepressors mSin3A and HDAC2 and reduction of histone H3 and H4 acetylation at the promoter.^39^ Moreover, SWI/SNF complex components can be differentially recruited by TFs for activation or suppression at the same promoter. For instance, in primary T lymphocytes under basal conditions, hBRM is recruited by native STAT1 to the IFNγ-activated sequences (GAS) of the *Hsp90α* gene in a mSin3/HDAC corepressor complex. Upon IFNγ stimulation, hBRM is acetylated by p300 and dissociates from the corepressor complex, allowing STAT1 to become phosphorylated and recruit BRG1 to the GAS, leading to elevated gene transcription.^40^ Some SWI/SNF subunits perform specialized functions in hematopoiesis. For instance, BCL11A is indispensable for normal HSC function. BCL11A deficiency results in HSC defects with an aging-like phenotype and cell cycle delay caused by decreased Cdk6 expression.^41^ BCL11A is also required for normal lymphopoiesis. Bcl11a mutant embryos lack B cells and the expression of the downstream TFs Ebf1 and Pax5 and have alterations in several types of T cells.^42^ Bcl11b acts directly upstream of Gfi1 to maintain its expression in mature type 2 innate lymphoid cells.^43^ Moreover, BRG1 regulates T helper 2 (Th2) differentiation and Th2 cytokine transcription via its recruitment to the Th2 locus control region (LCR) by STAT6 and NFAT.^44^ During myeloid differentiation, RUNX1 interacts with subunits BRG1 and INI1, which supports binding of the SWI/SNF complex to RUNX1 target genes related to the myeloid lineage and generates active histone modifications.^33^ An interaction between C/EBPβ and hBRM is essential for activating the myeloid *mim-1* gene in HD3 erythroblasts.^35^ During macrophage differentiation, ATF-3 associates with BRG1 and β-actin to transactivate the SLC11A1 promoter by initiating Z-DNA formation.^34^ BAF60B controls the differentiation of neutrophil granulocytes via recruitment of CEBPε to the promoters of neutrophilic granule genes. Adult mice with BAF60B-deficient hematopoietic cells develop myelodysplasia and excess blasts.^45^ Furthermore, inhibition of ACTL6A promotes granulocytic differentiation in NB4 and HL-60 cells via decreasing its interaction with the TF Sox2.^46^

Cancer genome sequencing has revealed that SWI/SNF subunits are the most frequently mutated (~20%) CRC in hematological malignancies. Inactivating mutations of *BRG1/SMARCA4*, *SNF5/SMARCB1*, *BCL11B*, and the BAF complex subunits *SMARCA2* and *ARID1A* are prevalent in leukemia. The presence of BRG1 is critical for the oncogenic Myc expression of leukemia cells because it maintains TF occupancy at critical Myc enhancers and enables long-range chromatin looping interactions with the Myc promoter. Notably, these Myc enhancers coincide with a region that is focally amplified in ~3% of AML. BRD9 also supports AML cell proliferation and an undifferentiated cell state by sustaining enhancer-mediated Myc expression using its bromodomain, which mediates acetyl-lysine histone H3 recognition. Inhibition of the BRD9 bromodomain selectively suppresses the proliferation of human AML cell lines. These findings define a leukemia maintenance function of SWI/SNF that is linked to enhancer-mediated gene regulation^47–49^ (Fig. 3). In addition to homozygous inactivation, chromosomal translocations and haploinsufficiency for SWI/SNF subunits are also involved in leukemia. For example, the *BCL11B* translocation t(5;14)(q35;q32.2) is present in 20–25% of pediatric and up to 5% of adult T-ALL and defines a molecular subset of this malignancy. Fifty percent of T cell prolymphocytic leukemias display deletions at 22q11, the location of *SNF5*. Additionally, conditional deletion of *SNF5* in mice leads to T cell lymphomas with short latency and 100% penetrance.^50^ Generally, specific mutations or translocations affecting SWI/SNF subunits may produce gain-of-function properties that result in an oncogenic SWI/SNF complex. In contrast, the inactivation or loss of some SWI/SNF subunits potentially affects TF activity,^51^ global histone modification^52^ or cooperating pathways,^53^ contributing to malignant hematopoiesis.

### ISWI complex

The imitation switch (ISWI) is an evolutionarily conserved CRC that contains SNF2H/SMARCA5 or SNF2L/SMARCA1 as the ATPase subunit and a C-terminal SANT domain adjacent to a SLIDE domain (SANT-like ISWI), which together form a nucleosome recognition module. ISWI mediates DNA accessibility by catalyzing nucleosome spacing and sliding reactions without displacing the histone octamer from DNA. In mammals, seven different ISWI complexes have been described, including RSF, ACF/WCRF, CHRAC, WICH, NoRC, CERF, and NuRF. Among them, SNF2L is found in the NURF and CERF complexes, and SNF2H is found in the CHRAC, NoRC, ACF, RSF, and WICH complexes. To date, except for SNF2H, the identities of other components of this complex in hematopoiesis remain largely unknown. Zikmund et al. found that SNF2H regulates murine early T cell development by guiding the transcription of early differentiation programs at the DN3 stage, and SNF2H deficiency leads to thymocyte proliferation and survival defects via the activation of the DNA damage response.^54^ SNF2H also plays indispensable roles in the maturation of definitive HSCs and the completion of erythropoiesis. SNF2H is abundantly expressed in mammalian early HSCs and proliferating progenitors, whereas its levels are downregulated during erythroid terminal differentiation.^55^ Knockout of murine SNF2H abrogates definitive hematopoiesis within the fetal liver, causing anemia and lethality at E18.5. Inhibition of SNF2H levels significantly suppresses cytokine-induced erythropoiesis in human CD34+ progenitors and erythroid progenitor proliferation.^56^ Primary erythroid progenitor defects in SNF2H involved a slowed onset of definitive hematopoiesis and defective proerythroblast-to-basophilic erythroblast maturation, marked by reduced expression of GATA-1 and a defect in hemoglobin switching.^57^ Interestingly, SNF2H appears to interact with GATA-1 in mouse proerythroblast/basophilic erythroblastic MEL cells, suggesting that SNF2H and GATA1 may cooperate in erythroid differentiation.^58^ Furthermore, Dluhosova et al. found that in normal myeloid cells, the association of SNF2H with the cohesin complex facilitates CCCTC-binding factor (CTCF) binding to the imprinting control region (ICR) of the *H19/Igf2* genes and an upstream regulatory element (URE) of the PU.1 gene, where SNF2H regulates the transcriptional outcome of CTCF as an enhancer-blocking cofactor.^59^

SNF2H was also found to be upregulated in CD34+ AML progenitors. After complete hematologic remission, SNF2H levels decreased, suggesting that overexpression of SNF2H may dysregulate the genetic program required for normal differentiation.^55^ Moreover, the SNF2H/CTCF binding site at the *PU.1* gene was methylated in AML, which prevented SNF2H/CTCF binding. Upon demethylation by 5-azacitidine (AZA) treatment, SNF2H/CTCF occupancy was partially restored, accompanied by decreased PU.1 expression and induced myeloid differentiation, suggesting that recruitment and occupancy of the SNF2H/CTCF complex is critical for normal myelopoiesis^59^ (Fig. 3).

### NuRD/Mi-2 complex

The NuRD/Mi-2 complex is a multifunctional epigenetic regulator that contains ATPase/helicase Mi-2, HDAC-1/2, Metastasis-associated Protein 1-3 (MTA1-3), methyl binding protein 3 (MBD3), and retinoblastoma-associated protein 46 and 48 (RbAp46/48). The NuRD/Mi-2 complex is itself part of a larger protein complex called the MeCP1 complex. The latter contains the additional components MBD2, P66, and P68.^60^ NuRD/Mi-2 functions primarily as a corepressor complex that induces chromatin compaction via the coupling of nucleosome sliding and HDAC activities and associates with methylated DNA. It can also activate gene transcription at specific loci. For example, GATA-1 interacts with NuRD/MeCP1/HDAC1 complexes through cofactor FOG-1. This complex broadly occupies GATA-1/FOG1-activated genes (i.e., *β-globin* and *Ahsp*) and GATA-1/FOG1-repressed genes (i.e., *GATA-2*, γ-globin, *c-myc*, *c-kit*, and *Hes1*) in erythroid cells and megakaryocytes (MKs), depending on the transcriptional and cellular context.^61^ The normal cooperation of the NuRD complex with other cofactor components is critical to the function of TCs. For example, the ability of FOG1 to augment GATA-1-induced transcription requires NuRD binding. Knock-in mice bearing three adjacent point mutations (R3G, R4G, and K5A) within the NuRD-binding module of FOG-1 that abrogate NuRD binding displayed thrombocytopenia and anemia with splenomegaly and extramedullary hematopoiesis.^61^ Furthermore, mice harboring disrupted FOG1/NuRD interaction (*Fogki/ki*) produced *Fogki/ki* CMPs and *Fogki/ki* MEPs that gave rise to significantly fewer and more immature MKs and erythroid colonies while retaining multilineage capacity to differentiate into Mast cells (MCs) and other myeloid lineage cells in vitro. In particular, the NuRD/MeCP1 complex is required for GATA-2 and non-Meg/E gene silencing during early erythroid differentiation, suggesting that the FOG1/NuRD interaction is not only required for erythropoiesis and megakaryopoiesis but also associated with suppression of alternative lineage choices.^62^

The NuRD/Mi-2 complex has been implicated in regulating both B and T cell differentiation. Prior to B-lineage specification and commitment, the MBD2/NuRD complex inhibits transcription of the B cell-specific *mb-1* gene via cooperative interactions between the major domains of Mi-2β with *mb-1* promoter chromatin and the binding of methylated promoter CpGs by MBD2.^63^ Specifically, SWI/SNF and NuRD/Mi-2 complexes function in opposition to enable or limit activation of the *mb-1* promoter by the TFs EBF and Pax5 during B cell development, suggesting that the NuRD/Mi-2 complex acts as a ‘gatekeeper’ by maintaining a high threshold for transcriptional activation.^64^ The MBD3/NuRD complex controls B versus T lineage fate and prevents the B cell commitment-associated transcriptional program by restricting Ebf1 transcriptional activity.^65^ Additionally, depletion of MTA3 in early thymic T cell progenitors leads to compensatory hyperproliferation of immature thymocytes and development of T cell lymphoma.^65^ MTA3 interacts with a transcriptional repression domain of BCL-6 that is required for BCL-6-dependent transcriptional repression. Their interaction is regulated by acetylation of BCL-6. Inhibition of NAD-dependent deacetylation by niacinamide, which results in the accumulation of the acetylated form of endogenous BCL-6, impairs its interaction with MTA3. Upregulation of BCL-6 and MTA3 in human plasma cell lines reprograms the cells to express markers of earlier stages of B lymphocytes,^66^ suggesting that the MTA3–BCL6 complex has a prominent role in B cell fate determination. MTA2 is critical for B cell maturation in the BM and spleen. Mechanistically, AIOLOS/IKAROS recruits the MTA2/NuRD complex to repress pre-BCR genes, such as *Igll1* and *VpreB1*, in pre-B cells via regulation of H3K27 acetylation. MTA2 also cooperates with OCA-B to regulate the pre-B cell to immature B cell transition via repressing the *Igll1* and *VpreB1* genes.^67^

The ATPase/helicase Mi-2β is essential for several steps in T cell development, including the double-negative (DN) to double-positive (DP) transition, the developmental expression of CD4 and the proliferation of mature T cells.^68^ In differentiating DP thymocytes, Mi-2β activates CD4 expression via recruitment of p300 and the E box-binding protein HEB to the CD4 enhancer, which causes histone H3 hyperacetylation of this regulatory region.^68^ NuRD/Mi-2β could determine the transcriptional activity of TFs in a stage-specific manner. Naito et al. found that in DN thymocytes, Ikaros binding to the CD4 silencer contributes to its repressive activity through interactions with HDACs and associated chromatin-remodeling complexes, such as the SWI–SNF complex. In DP thymocytes, concomitant binding of Mi-2β with Ikaros to the CD4 silencer antagonizes the repressive activity of Ikaros via the recruitment of HATs, i.e., MOZ and TAFII250, which allows for CD4 expression. This case suggests that concomitant interactions between functionally opposing chromatin-regulating machinery are an important mechanism of gene regulation during lineage determination.^69^ Interestingly, in T cells, a majority of Ikaros exists in the Mi-2/NuRD complex. A small fraction of Ikaros is found in association with SWI/SNF in a distinct complex. The locations of Ikaros/Mi-2/NuRD and Ikaros/SWI/SNF are differentially distributed during the cell cycle, implying that one TF could target distinct transcriptional machinery in a context-dependent manner.^70^ Furthermore, the modification status of TFs could affect TC activity via the recruitment of different cofactor components. For instance, during primary CD4+ T cell activation, PKC-mediated BCL11B phosphorylation switches BCL11B from a repressor to an activator of the *IL-2* gene by disrupting interactions between BCL11B and the MTA/NuRD complex and recruiting p300.^71^

The NuRD complex plays a key role in leukemogenesis (Fig. 3). In APL, the fusion protein PML-RARα binds and recruits the NuRD complex to target genes, such as the tumor suppressor *RARβ2*, which allows recruitment of Polycomb repressive complex 2 (PRC2) and DNMT3a, leading to promoter silencing. Knockdown of the NuRD complex not only prevents histone deacetylation and chromatin compaction but also impairs DNA and histone methylation, thus promoting cellular differentiation.^72,73^ In addition, the stem cell factor SALL4 contributes to AML leukemogenesis by recruiting the NuRD/HDAC complex to the *PTEN* and *SALL1* promoters and suppressing their expression. Targeting AML can be achieved by blocking the interaction between SALL4 and the NuRD/HDAC complex. Thus, enzymatic components of the NuRD/Mi-2 complex may provide novel targets for pharmacological manipulation.^74^ Alterations in the composition of the NuRD complex also contribute to leukemogenesis. Biswas et al. found that MBD3 deficiency relieves HDAC1-associated transcriptional repression in a locus-specific manner, which in turn results in increased occupancy of KDM6A, H3K27Ac, and CBP, leading to *DOCK5/8* expression and maintenance of the Rac GTPase program in AML cells.^75^

## Histone-modifying cofactors

Histone-modifying cofactors are epigenetic enzymes that add or remove chemical residues from the histones they modify. Generally, HATs transfer acetyl groups from acetyl-CoA to lysine residues of histone proteins, which opens chromatin and allows gene transcription, whereas HDACs have a repressive effect on gene expression by deacetylating lysine residues on histone tails. Histone methylation is the methylation of lysine or arginine residues. Methyl marks are created by HMTs, so-called “writers,” and removed by histone demethylation enzymes, termed “erasers”.

### Histone acetyltransferases

HATs typically exist in multiprotein complexes where they execute acetylation programs that acetylate not only histone tails but also nonhistone proteins, which modulates TC DNA binding or transcriptional activity. For example, in eukaryotic cells, CBP/p300 is a component of multiprotein complexes composed of the basal transcriptional apparatus (TFIIB, TBP, and PolΙΙ), specific TFs, acetyltransferases (PCAF, GCN5) and other coactivator complexes (ARC, etc.).^76^ As an essential coactivator, CBP/p300 associates with large numbers of TFs in virtually all hematopoietic lineages. In stem/multipotent progenitor cells, CBP/p300-mediated acetylation of GATA-2 enhances its DNA binding and transactivation activities and suppresses GATA-2-mediated growth inhibition.^77^ Moreover, the GATA2–CBP complex induces GATA2 positive autoregulation.^78^ In committed progenitor/differentiated cells, GATA1-dependent displacement of the GATA2–CBP complex is involved in the GATA switch.^78^ Upon differentiation of erythroid cells, TFs, including EKLF,^79^ GATA1,^80^ NF-E2,^81^ and SCL/Tal1,^82^ associate with CBP/p300 or p300/CBP association factor (P/CAF) to promote normal erythropoiesis via modulating *globin* gene expression. Some TFs are themselves subject to acetylation by CBP/p300 or P/CAF, which increases TF transcriptional activity^83^ or DNA-binding activity^82,84^ and, in some cases, selectively stabilizes or destabilizes TF-associated CRCs.^82^ An important role of CBP/p300 in establishing lymphoid and myeloid differentiation is supported by their interactions with TFs, including RUNX1/AML1,^85^ PAX5,^86^ C/EBPβ,^87^ BCL11B,^88^ T-bet,^89^ c-Myb,^90^ Bcl-6,^91^ and the Ets family (Ets-1 and PU.1).^92,93^ These TFs are critical for myeloid-specific or lymphoid-specific gene transcription due to their regulation of the enzymatic activity of CBP/p300. For example, NF-E2, C/EBPβ, and PU.1 have been shown to inhibit^93^ or facilitate^86,87,94^ CBP/p300-mediated nucleosomal HAT activity.

**Fig. 4:**
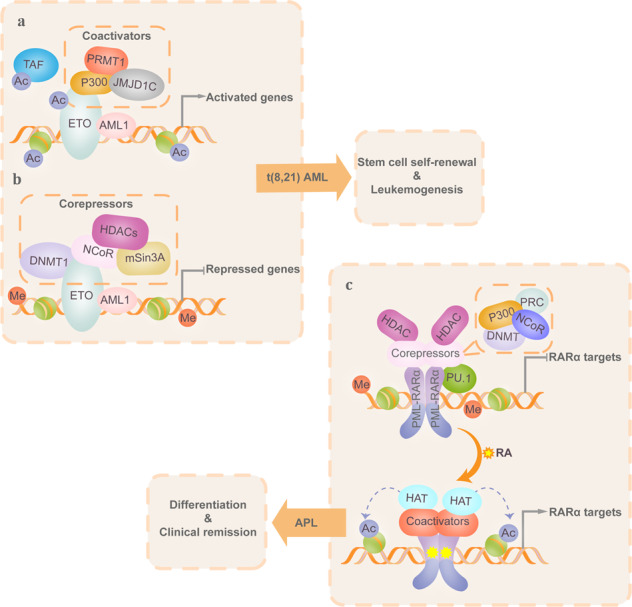
Aberrant recruitment of HAT/HDAC-containing complexes is a general mechanism of leukemogenesis. Chromatin accessibility plays a critical role in regulating cell type-specific gene expression during hematopoiesis but has also been suggested to be abnormally regulated during leukemogenesis. Oncogenic TF or fusion protein binding was found at accessible chromatin regions with aberrant recruitment of the coactivator (CoA) or corepressor (CoR) complexes that affected histone modification patterns, altered the chromatin structure and thereby facilitated DNA accessibility. Furthermore, in some cases, HATs/HDACs are critical to the optimal oncogenic activity of leukemia TFs or fusion proteins via regulating their acetylation levels. Therefore, disrupting the balanced interplay of the epigenetic environment and oncogenic TFs or fusion proteins might be used as a therapeutic strategy. **a** Upper panel, P300-containing CoA complexes contribute to the self-renewal and leukemogenesis of HSPCs through acetylating the AML1-ETO fusion, which promotes its transcriptional activation and histone H3. **b** The AML1-ETO fusion recruits CoR complexes consisting of the nuclear receptor corepressor (N-CoR), HDAC1, SIN3 transcription regulator family member A (mSin3A), and DNA methyltransferase 1 (DNMT1), which repress gene transcription by enzymatic deacetylation of histones, DNA methylation, and creation of a repressive chromatin structure. **c** In APL blast cells, the PML-RARα fusion forms oligodimers and binds DNA through recruitment of the CoR–HDAC complex, which leads to deacetylation of histones and H3K27/H3K9 methylation and subsequently produces a condensed chromatin structure that represses the transcription of target genes. While all-trans retinoic acid (RA) or arsenic trioxide (ATO) mediates degradation of PML-RARα, which is replaced by the RARα/RXR heterodimer, and converts the CoR-HDAC into a CoA–HAT complex that reactivates gene transcription and restores differentiation

Aberrant lysine acetylation has been implicated in malignant hematopoiesis, and mice that lack p300 and/or CBP display a high incidence of hematological malignancies, including histiocytic sarcomas and myelogenous and lymphocytic leukemia. Mutations in the *CBP* and *p300* genes have been identified in non-Hodgkin lymphoma and relapsed ALL patients.^95^ These mutations generally affect the HAT domain, reducing CBP acetyltransferase activity, which leads to impaired histone acetylation and diminished expression of target genes. In this case, reactivation of CBP/p300 has emerged as a potential therapeutic strategy.^96^ Some oncogenic TFs, such as C-myb, EVI1, E1A, and E2A, perform their transforming activity by relying on CBP or p300.^84,97–99^ HAT-containing fusion proteins (CBP-MOZ, CBP-MLL, CBP-MORF, MOZ–TIF2, and MOZ–p300) caused by chromosomal translocations also contribute to leukemogenesis. CBP fusions generate powerful chromatin-modifying activity, leading to transcriptional deregulation by promoting histone acetylation of genomic regions. MOZ fusions lead to repressed differentiation, hyperproliferation and self-renewal of myeloid progenitors through deregulation of MOZ-regulated target gene expression.^100^ Furthermore, a portion of oncofusion proteins perform their function via recruiting HATs (Fig. 4). For example, in t(8;21) AML, p300 acetylates AML1-ETO and potentiates its transcriptional activation, which enhances the leukemogenicity of HSPCs. Inhibition of p300 could reverse the acetylation of AML1-ETO and leukemic transformation.^101^ In APL and AML, AML1-ETO and PML-RARα display similar binding sites where both create a hypoacetylated chromatin environment at accessible p300-binding sites, suggesting that the chromatin-modifying characteristics of HATs can be used to predict fusion protein-binding sites.^101–103^

### Histone deacetylases

HDACs are widely involved in the development of various hematopoietic lineages, together with a variety of TFs. For example, in HSCs, HDAC3 cooperates with Ncor2 to repress the fos-vegfd cascade by modulating the acetylation level of the fos promoter, thereby stimulating HSC formation.^104^ HDAC3 can also directly interact with GATA2 and repress GATA2-dependent target genes by modifying the acetylation status of GATA2 in HPCs.^105^ In the granulocyte–monocyte lineage, HDAC4 promotes monocyte and CD8α(+) conventional DC differentiation via the reduction of the acetylation of both histone 3 and STAT6 and the subsequent transcriptional activation of Arg1.^106^ HDAC1 and HDAC7 are involved in terminal macrophage differentiation via their interaction with MEF2A/D heterodimers, which represses c-Jun.^107^ The reduced interaction of Runx1 with HDAC1/HDAC3 induced by Src kinase-mediated Runx1 phosphorylation is related to increased DNA affinity and the induction of granulopoiesis.^108^ Furthermore, Runx1 and PU.1 independently interact with the ETO2–SIN3A–HDAC2 corepressor complex and coactivate the expression of M-CSFR and GM-CSFR, which control the development and activation of granulocyte–monocyte cells.^109^ For the erythrocyte lineage, HDAC1/Sin3A can be recruited by EKLF to inhibit β-globin expression in undifferentiated EBHX11L cells, while this complex can be converted to the EKLF-p300/CBP-SWI/SNF complex and promote β-globin expression during the differentiation of EBHX11L cells to a primitive erythroid phenotype.^110^ HDAC-containing TCs are also implicated in γ-globin to β-globin switching, highlighting that HDACs in association with *globin* gene switching may provide molecular targets for intervening in *β-globin* gene disorders.^111^ The function of class II HDACs, which shuttle TFs between the cytoplasm and nucleus, is critical for erythropoiesis. For instance, although HDAC5 does not have deacetylase activity, it could shuttle GATA1 and EKLF from the cytoplasm to the nucleus via the formation of an erythroid-specific HDAC complex.^112^ For the lymphocyte lineage, HDAC3 was identified as a component of the STAT5a–LSD1 complex in pro-B cells, where it plays dual roles in determining the activation or repression of STAT5a-targeting genes based on protein interactions, genomic-binding localization and binding affinities.^113^ In mature B cells, Bach2 recruits the HDAC3–NCoR1/NCoR2–Rif1 complex to repress Prdm1 transcription by deacetylating histone H3-K9, impeding the terminal differentiation of B cells into plasma cells.^114^ Moreover, HDAC7 specifically interacts with MEF2C in pro-B cells, and a HDAC7–MEF2C complex is involved in silencing lineage-inappropriate genes, ensuring correct B cell differentiation.^115,116^

HDACs are critical for the optimal oncogenic activity of leukemia fusion proteins (Fig. 4). For example, AML1-ETO and RARα-PLZF cause transcriptional repression of genes responsible for hematopoietic differentiation via recruitment of HDAC1/3, thus contributing substantially to leukemogenesis.^117,118^ Interactions with different HDACs confer different functions to TFs. For example, in B cell non-Hodgkin lymphoma (B-NHL), aberrant expression of the HDAC9–BCL6 complex contributes to lymphomagenesis.^119^ In follicular lymphoma (FL) and diffuse large B cell lymphoma (DLBCL), CBP mutations disable the acetylation of the HDAC9–BCL6 complex and lead to unopposed deacetylation by the BCL6–SMRT–HDAC3 complex at enhancers of B cell immune response genes, which promotes lymphomagenesis.^120^ In miR-155-induced pre-B cell leukemia/lymphoma, HDAC4 in complexes with BCL6 plays an important role in suppressing leukemogenesis.^121^

### Histone methyltransferases

Histone methylation signatures serve as an important regulatory mechanism in hematopoiesis. In HSCs, numerous genes involved in the differentiation of multiple lineages are marked by bivalent marks that display the repressive mark H3K27me3 and the active marks H3K4me2 and H3K4me3. Many lineage-specifying genes in these primitive cells possess a diverse range of histone modification patterns, suggesting that specific epigenetic combinations prepare these genes for selective expression or silencing during lineage commitment.

HMTs are implicated in hematopoiesis regulation via the following multilayer mechanisms. (1) Specific regulation of TF methylation based on the developmental stage of the cell. For instance, Runx1 is arginine-methylated by PRMT1 upon myeloid differentiation but by PRMT4 in HSPCs. The former prevents RUNX1 association with the SIN3A corepressor and functions as a coactivator for RUNX1-dependent transcription.^122^ The latter blocks myeloid differentiation by assembling a Methyl-RUNX1-dependent repressor complex that inhibits RUNX1 target genes.^123^ (2) Specific modification of histone marks via HMT-containing TCs. In megakaryocytic/erythroid progenitors, a RUNX1–PRMT6–Sin3A–HDAC complex has an impact on bivalent histone marking at megakaryocytic differentiation genes. This corepressor complex is exchanged with a RUNX1 coactivator complex following megakaryocytic differentiation.^124^ (3) Regulation of TF expression via proteasome-mediated degradation. For example, MLL stabilizes RUNX1/AML1 from ubiquitin–proteasome-mediated degradation by masking the RUNX1–proteasome interaction domain and possibly by methylating lysine residues. In MDS and AML, a subset of mutations at the N terminus of RUNX1 disrupt its interaction with MLL, leading to loss of H3K4me3 marks within PU.1 regulatory regions and decreased PU.1 expression.^125^ (4) Crosstalk between HMTs and other TCs. For example, PRMT4 has been found to engage in crosstalk with Mi2α/NuRD,^126^ SWI/SNF, and Mediator complexes to regulate hematopoietic signaling.^127^ Crosstalk between PRMT1 and the E3 ligase CNOT4 on RBM15 controls the RNA splicing of some hematopoietic TFs.^128^

**Fig. 5:**
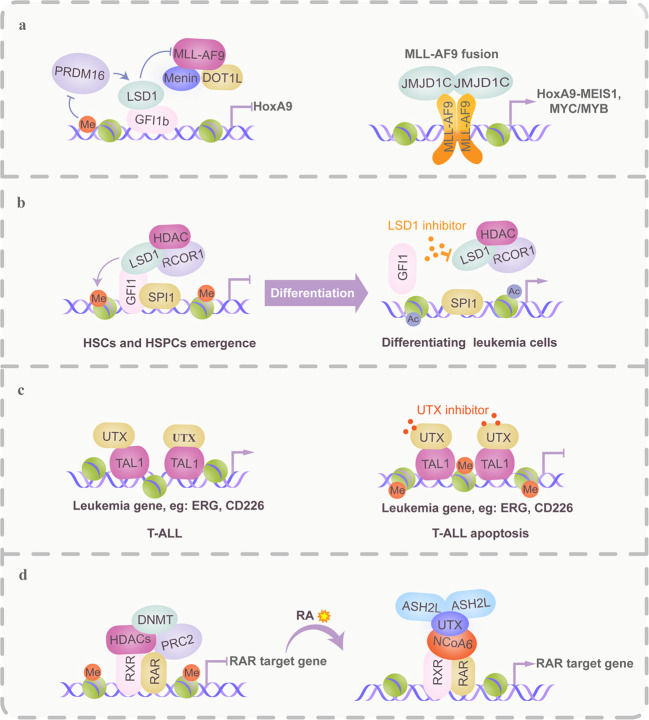
HMT/HDT-based regulatory mechanisms in TCs are implicated in the pathogenesis and treatment of malignant hematopoiesis. HMTs and HDTs are recurrently mutated or aberrantly expressed in a variety of hematological malignancies. Their intrinsic activities on histone and nonhistone proteins within transcriptional complexes are critical for epigenetic control in normal and malignant hematopoiesis and are amenable to drug intervention or involved in drug therapeutic effects. **a** Left panel in mixed lineage leukemia (MLL)-AF9 fusion leukemia. MLL-AF9 recruits DOT1L, a histone 3 lysine 79 methyltransferase (H3K79me1/me2/me3), which leads to hematopoietic transformation via H3K79 dimethylation that causes aberrant transcription of genes such as HOXA9 and MEIS1. PRDM16 (a histone H3K4 methyltransferase), an antagonist to DOT1L, activates Gfi1b-mediated gene transcription, which in turn downregulates the HOXA gene cluster. However, PRDM16 expression is always silenced by DNA methylation in MLL-AF9 leukemia. Right panel, similar to DOT1L, the H3K9me2/me1 demethylase JMJD1C contributes to MLL-AF9 leukemia maintenance by affecting MYB, MYC, and HOXA9-MEIS1 gene expression programs, suggesting that individual genes can be regulated by different kinds of HMTs and/or HDT-containing TCs. **b** Growth factor independence 1 (GFI1) is critical to the initiation of the endothelial-to-hematopoietic transition (EHT) due to its recruitment of the LSD1–CoREST repressive complex to epigenetically silence the endothelial program and allow the emergence of HSCs. Disruption or separation of the GFI1–LSD1 repressive complex by LSD1 inhibitors is considered to induce differentiation in certain subtypes of AML. **c** UTX, a coactivator of TAL1, is essential to leukemia maintenance in TAL1-positive cells due to its promotion of an open chromatin configuration at target gene sites by H3K27me3 demethylation. A therapy based on UTX inhibition is efficient at inducing cell death through downregulation of the TAL1 leukemic gene expression program. **d** UTX is a critical mediator of RA-induced differentiation in leukemic cells. RA treatment leads to the coordinated removal of repressive marks, the displacement of polycomb group proteins, and the deposition of activating marks. RA promotes the activation of RAR target genes by recruiting NCoA6, UTX, and ASH2L, concomitant with the demethylation of H3K27 and trimethylation of H3K4

The aberrant recruitment of HMTs to key hematopoietic genes by oncofusion TFs is critical to transcription deregulation. The SET domain that is required for H3K4 methyltransferase activity is consistently lost in MLL fusions and is often compensated by interaction with alternative HAT/HMT enzymes through partner proteins. For example, the MLL–EEN fusion protein recruits CBP and PRMT1 to induce the transformation of primary myeloid progenitors via introduction of aberrant H4R3me2 at MLL target *Hox* loci.^129^ Moreover, MLL fusion partners, such as AF4, AF9, AF10, and ENL, associate with DOT1L, which results in activation of H3K79me2, aberrant transcriptional elongation by recruitment of DOT1L to the Pol II elongation complex, and maintenance of an open chromatin state.^130^ A graded reduction of DOT1L recruitment to MLL-AF9 leads to differential loss of H3K79me2 and H3K79me3 at MLL-AF9 target genes.^130^ Interestingly, PRDM16 specifically suppresses MLL fusion protein-induced leukemogenesis via activation of Gfi1b, which in turn inhibits HOXA gene clusters^131^ (Fig. 5). Thus, methyltransferases may be promising targets for epigenetic treatment.^132^ In particular, deregulation of H3K27 methylation is associated with tumorigenesis. In this respect, gain-of-function mutations or upregulation of the H3K27 methyltransferase EZH2 have been found in FL, germinal center B cell lymphomas, MDS and AML. The EZH2 inhibitor 3-deazaneplanocin A (DZNep) selectively induces apoptosis in various hematological malignancies and promotes erythroid differentiation in K562 cells.^133^

### Histone demethylases

HDTs are mainly categorized into two families: the LSD family (LSD1-2) and the JmjC family (KDM2-7). The LSD family contains flavin adenine dinucleotide-dependent monoamine oxidases, which erase methyl marks at the H3K4 and H3K9 residues. The JmjC family contains Fe(II)-dependent and α-ketoglutarate-dependent dioxygenases, which can be further divided into groups with H3K4-, H3K9-, H3K27-, or H3K36-demethylating activities based on the structure of the JmjC domain.

HDTs are extensively involved in hematopoietic differentiation, ranging from embryonic to adult hematopoiesis, via HDT-TF interplay. For example, the LSD1-mediated downregulation of the *etv2* gene in hemangioblasts is essential for the initiation of endothelial to hematopoietic transdifferentiation (EHT).^134^ In HSPCs, SALL4 dynamically controls the binding levels of LSD1 to hematopoietic regulatory genes, and LSD1 contributes to the repressive effects of SALL4 by affecting local chromatin structure.^135^ Specifically, lineage-restricted deployment of the LSD1/CoREST complex controls hematopoietic differentiation. For example, GFI1 recruits the LSD1/CoREST complex to epigenetically silence the endothelial program in the hemogenic endothelium and allow the generation of HSCs.^136^ In erythropoiesis, the LSD1/CoREST–BCL11A complex is required for full developmental silencing of mouse embryonic *β-like globin* genes and human *γ-globin* genes in adult erythroid cells.^137^

The TF-HDT interplay is multifaceted. As interacting cofactors, HDTs generally act as coactivators of oncogenic TCs. For example, UTX is a coactivator of TAL1 and removes H3K27me at the TAL1 target locus, allowing TAL1-mediated activation of a leukemic program.^138^ JMJD1C functions as a coactivator for the AML1-ETO-containing AETFC complex to drive leukemic programs by maintaining low H3K9me2 levels.^139^ JMJD1C is also an important mediator of MLL-AF9-driven and HOXA9-driven leukemia stem cell (LSC) function (Fig. 5). Loss of JMJD1C significantly reduces the LSC frequency in MLL-AF9 and HOXA9/MEIS1 leukemias.^140^ In this respect, some HDTs binding to oncogenic TFs have been suggested as drug targets (Fig. 5). For example, a ZEB2–LSD1 interaction is essential for the survival of T-ALL cells with high ZEB2 levels, which are sensitive to LSD1 inhibitors.^141^ Moreover, some inhibitors were developed to interfere with LSD1–GFI1/CoREST complex functions, including blockade of its scaffolding and enzymatic functions or displacement of LSD1 from the GFI1 repressor complex.^142,143^ However, TF-HDT interplay is subject to regulation by protein modifications. Li et al. found that PKA-mediated serine 172 phosphorylation of TAL1 modulates the transcription of target genes by dynamically recruiting the LSD1 complex, which is essential for hematopoietic progenitor differentiation and leukemogenesis.^144^ Furthermore, in many cases, cofactors could simultaneously affect oncogenic TFs and tumor suppressor programs.^145^ Specifically, TFs and cofactors mutually regulate each other’s expression levels via regulation loops. For example, ARID5B and TAL1 not only positively regulate each other’s transcription but also coordinately control TAL1 target genes, which contribute to T cell leukemogenesis.^146^ Furthermore, the interplay between cofactors plays a pivotal role in the optimal activity of TCs. For example, JMJD1B displayed histone H3K9me1/2 demethylase activity and induced leukemogenic lmo2 expression via a synergistic interaction with CBP.^147^

HDTs are involved in drug response (Fig. 5). For example, in APL, a UTX–NCoA6–ASH2L–PML/RARα complex is essential for APL cell differentiation upon RA treatment via H3K4 methylation, H3K27 demethylation, and the consequent transcriptional activation of RAR target genes.^148^ Similarly, PHF8, as a coactivator, is recruited by RARα fusions to activate the expression of their downstream targets. Forced expression of PHF8 resensitizes ATRA-resistant APL cells.^149^ In contrast, TFs are also involved in the antileukemic effects of HDT inhibitors. Regarding this subject, Cusan et al. revealed that recruitment of a myeloid TF network regulated by C/EBPα and PU.1 mediates the antileukemic activity of an LSD1 inhibitor. Perturbation of C/EBPα and PU.1 occupancy at LSD1 inhibitor-induced dynamic sites confers to AML cells increased resistance to LSD1 inhibition.^150^

## Conclusions

Gene transcription determines cellular phenotype and function, and TFs play a key role in controlling gene expression profiles in response to cellular signals. However, the transcription process involves the interaction of TFs with not only the DNA sequence but also the cofactors within TCs. Cofactors modulate histone affinity for DNA and chromatin accessibility to their cognate-binding proteins by compaction of DNA/histone complexes. Their biochemical and molecular characteristics greatly impact the activity of TCs. Importantly, cofactors can have catalytic/noncatalytic^145^ and histone/nonhistone effects on hematopoietic cells, which confers to cofactors the ability to regulate a variety of cellular events in normal and malignant hematopoiesis. Cofactor–TF interactions are gene-dependent or context-dependent, as reflected by the following characteristics: (1) Different genes regulated by the same TF may recruit different cofactors. (2) One cofactor could act as a coactivator or corepressor on different genes and utilize different domains to act on interacting TFs. (3) Cooperation of one cofactor with different TFs or TCs may produce distinct developmental or leukemic properties.^151^ The interplay between TFs and cofactors is multifaceted and involves direct and indirect regulatory mechanisms, which depend on the molecular properties of cofactors and can include the following: (1) Organization of TCs with scaffolding proteins. (2) Regulation of the transcriptional activity, DNA-binding capacity or protein modification of TFs. (3) Involvement in modifying the chromatin landscape. (4) Cooperation between cofactors within TCs, which is a prerequisite for TF transcription programs. TFs are important for guiding cofactors to particular genomic locations to modify the epigenetic environment. In some cases, TFs and cofactors could affect each other’s expression, activity, or binding states via signaling cascades, intermediate TF-mediated regulatory loops or crosstalk effects. For example, phosphorylation of RUNX1 by ERK enhances its transactivation activity by disrupting its interactions with mSin3A. TFs have also been found to mediate crosstalk between cofactors. For example, the recruitment of HDAC1 by Ikaros is critical for the repression of the demethylase KDM58.^152^

**Fig. 6:**
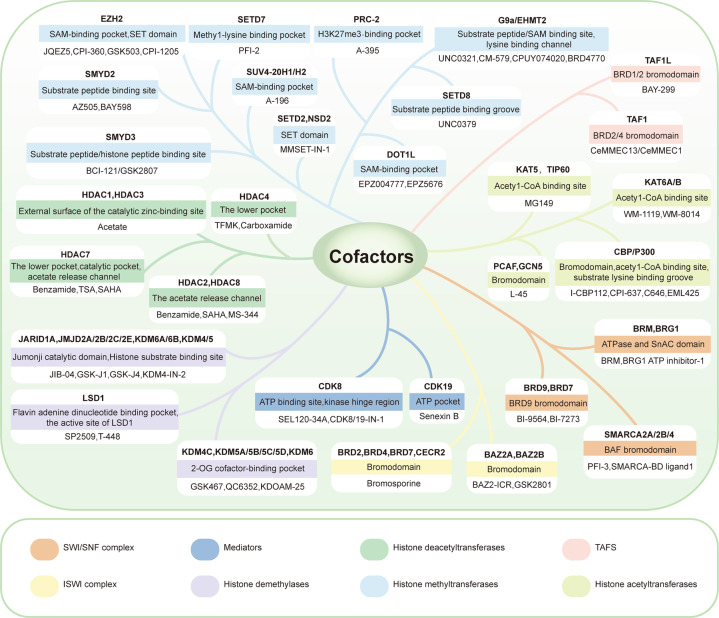
The targeted domains/sites used to design cofactor inhibitors. Abnormal epigenetic changes are amenable to pharmacological intervention. The emergence of cofactors as oncology targets has spurred significant drug discovery efforts with the goal of identifying small-molecule inhibitors that target their enzymatic sites, binding pockets, and protein/TF interactions for therapeutic applications. Binding sites and representative drugs for each kind of cofactor are shown

One hallmark of hematological malignancies is somatic mutations and genetic rearrangements of cofactors that impact the activity and expression of cofactors or the composition of TCs that cause alterations in signal transduction and gene expression programs. In particular, the activity of oncogenic TFs and fusion proteins is reliant on their interactions with cofactors, which provides increased opportunities for intervention in the disease by targeting cofactors^153^ (Tables 2–4). To date, a variety of selective small inhibitors have been developed to target the functional or interacting domains of cofactors (Fig. 6). For example, the formation of complexes between the MLL-binding pocket of WDR5-MLL and the p30 isoform of C/EBPα represents a functional vulnerability that can be pharmacologically exploited by the small-molecule antagonist OICR-9429 to trigger differentiation and growth arrest in C/EBPα-mutant AML.^154^ Drug resistance and limited therapeutic efficacy are key issues that hinder the clinical application of cofactor inhibitors. The ability to combine multiple chemotherapeutic or immunoregulatory agents is considered a major breakthrough in the treatment of hematological malignancies. For example, HDAC inhibitors have shown synergistic or additive effects with proteasome inhibitors, hormonal therapy, tyrosine kinase inhibitors, DNA-hypomethylating agents, and immune checkpoint inhibitors in preclinical and clinical settings.^155^

**Table 2:** Transcriptional cofactors with high frequency of mutations in hematological malignancies

Disease	Gene	Mutation number	Case number with mutation	Percentage (total number)
ALL	SETD2	25	10	13.7 (73)
	KMT2D	15	14	19.2 (73)
	KDM6A	4	3	4.1 (73)
	MED12	3	3	4.1 (73)
AML	EZH2	24	22	3.5 (622)
	PHF6	21	18	2.9 (622)
	MED12	6	6	3.0 (200)
	EP300	5	5	2.5 (200)
	CHD4	4	4	2.0 (200)
	CREBBP	4	4	2.0 (200)
CLL	CHD2	27	27	5.0 (537)
	DDX3X	13	13	2.4 (537)
	ARID1A	2	2	1.9 (105)
	MED12	9	9	1.7 (537)
DLBCL	KMT2D	16	12	29.3 (41)
	CREBBP	14	14	24.1 (58)
	EZH2	11	11	20.8 (53)
	ARID1A	4	4	9.8 (41)
	ARID1B	87	83	8.3 (1001)
	SETD1B	88	83	8.3 (1001)
	SMARCA4	79	75	7.5 (1001)
	TAF1	4	4	7.5 (53)
	EP300	4	4	7.5 (53)
	SETD2	5	4	7.5 (53)
Mature B-cell malignancies	KMT2D	338	247	32.7 (755)
	CREBBP	229	180	23.8 (755)
	ARID1A	83	80	10.6 (755)
	EZH2	65	63	8.3 (755)
MM	NSD2	5	5	2.4 (205)
	BCL7A	4	4	2.0 (205)
	KMT2D	4	4	2.0 (205)
Myelodysplasia	EZH2	2	2	6.9 (29)
	PHF6	1	1	3.4 (29)
	DOT1L	1	1	3.4 (29)
	FBXO11	1	1	3.4 (29)
MN	EZH2	4	4	2.6 (151)
	PHF6	2	2	1.3 (151)
	EZH1	2	2	1.3 (151)
Non-Hodgkin Lymphoma	EZH2	4	4	28.6 (14)
	CREBBP	3	2	14.3 (14)
	KMT2D	2	2	14.3 (14)

All data in this table come from the TCGA database.

**Table 3:** Transcriptional cofactors with abnormal gene fusions in hematological malignancies

Disease	Gene	Fusion number	Case number with fusion	Percentage (total number)
ALL	KMT2A	56	56	67.5 (83)
	MLLT10	4	4	4.8 (83)
	ARID1B	1	1	1.2 (83)
	CREBBP	1	1	1.2 (83)
	SETD2	1	1	1.2 (83)
	MLLT1	1	1	1.2 (83)
AML	KMT2A	10	10	5.0 (200)
	MLLT10	8	6	3.0 (200)
	NSD1	4	3	1.5 (200)
	ELL	3	3	1.5 (200)
	MLLT3	2	2	1.0 (200)
	BCL11A	1	1	0.5 (200)
	CREBBP	1	1	0.5 (200)
	KAT6A	1	1	0.5 (200)
	KMT2C	1	1	0.5 (200)
	CDK8	1	1	0.5 (200)
	ARID1A	1	1	0.5 (200)
	KDM2B	1	1	0.5 (200)
DLBCL	ARID4B	1	1	1.9 (53)
	SETD2	1	1	1.9 (53)

All data in this table come from the TCGA database.

**Table 4:** Transcriptional cofactors with high frequency of abnormal copy numbers in hematological malignancies

Disease	Gene	Cytoband	Type of CNA	Case number with CNA	Percentage (total number)
ALL	MLLT3	9p21.3	DEL	76	9.9 (764)
	ARID3A	19p13.3	DEL	46	6.0 (764)
	CHD4	12p13.31	DEL	24	3.1 (764)
	ARID3C	9p13.3	DEL	23	3.0 (764)
	DOT1L	19p13.3	DEL	21	2.7 (764)
	KDM5A	12p13.33	DEL	20	2.6 (764)
	SETD4	21q22.12	AMP	18	2.4 (764)
	SETD2	3p21.31	DEL	17	2.2 (764)
	NSD2	4p16.3	DEL	16	2.1 (764)
	KDM5A	12p13.33	AMP	16	2.1 (764)
AML	CHD2	15q26.1	DEL	19	7.9 (240)
	KMT2A	11q23.3	AMP	12	6.3 (191)
	JARID2	6p22.3	DEL	9	3.8 (240)
	EZH2	7q36.1	DEL	9	3.8 (240)
	MLLT10	10p12.31	DEL	8	3.3 (240)
	KMT2C	7q36.1	DEL	6	3.1 (191)
	KAT6A	8p11.21	DEL	7	2.9 (240)
	SETDB2	13q14.2	DEL	7	2.9 (240)
	EZH1	17q21.2	DEL	7	2.9 (240)
	EZH2	7q36.1	DEL	5	2.6 (191)
	ARID3A	19p13.3	AMP	6	2.5 (240)
DLBCL	BCL11A	2q16.1	AMP	8	16.7 (48)
	PRDM16	1p36.32	DEL	4	8.3 (48)
	SETD2	3p21.31	DEL	3	6.3 (48)
	SMYD3	1q44	DEL	3	6.3 (48)
	JARID2	6p22.3	AMP	2	4.2 (48)
	ARID2	12q12	AMP	2	4.2 (48)
	SMARCA2	9p24.3	AMP	2	4.2 (48)
	SMARCD1	12q13.12	AMP	2	4.2 (48)
	PBRM1	3p21.1	DEL	2	4.2 (48)
	BCL7A	12q24.31	DEL	2	4.2 (48)
	KMT2D	12q13.12	AMP	2	4.2 (48)
	SETD1B	12q24.31	DEL	2	4.2 (48)
	KDM5C	Xp11.22	AMP	2	4.2 (48)
	KDM4C	9p24.1	AMP	2	4.2 (48)
	HDAC7	12q13.11	AMP	2	4.2 (48)

All data in this table come from the TCGA database.

The interplay between cofactors and TFs globally affects the gene regulatory network. Future studies will be needed to identify more regulatory factors that dysregulate TC expression or function and determine how their dysregulation contributes to the pathogenesis of hematological malignancies. In particular, a functional switch in the cofactor–TF interplay is critical to malignant transformation. Hence, resolving specific cofactor components, the crystal structure of cofactors, identification of cofactors-related the epigenetic mechanisms, downstream genes, TF/cofactors or TF/DNA interactive interfaces may contribute to develop novel inhibitors in a given hematologic disorder.
